# Synthesis of Ni@NiSn Composite with High Lithium‐Ion Diffusion Coefficient for Fast‐Charging Lithium‐Ion Batteries

**DOI:** 10.1002/gch2.201900073

**Published:** 2019-11-22

**Authors:** Hong Zhao, Junxin Chen, Weiwei Wei, Shanming Ke, Xierong Zeng, Dongchu Chen, Peng Lin

**Affiliations:** ^1^ Shenzhen Key Laboratory of Special Functional Materials and Shenzhen Engineering Laboratory for Advanced Technology of Ceramics College of Materials Science and Engineering Shenzhen University Shenzhen 518060 P. R. China; ^2^ School of Materials Science and Energy Engineering Foshan University Foshan 528000 China; ^3^ Department of Mechanical and Aerospace Engineering Hong Kong University of Science and Technology Hong Kong 999077 Hong Kong

**Keywords:** fast‐charging, ion diffusion coefficient, lithium ion batteries, Ni@NiSn composite, Sn–Ni

## Abstract

To solve the problems of fast‐charging of lithium‐ion batteries in essence, development of new electrode materials with higher lithium‐ion diffusion coefficients is the key. In this work, a novel flower‐like Ni@SnNi structure is synthesized via a two‐step process design, which consists of the fabrication of Ni cores by spray pyrolysis followed by the formation of SnNi shells via a simple oxidation–reduction reaction. The obtained Ni@SnNi composite exhibits an initial capacity of ≈693 mA h g^−1^ and a reversible capacity of ≈570 mA h g^−1^ after 300 charge/discharge cycles at 0.5 C, and maintains 450 mA h g^−1^ even at a high rate of 3 C. Further, it is proved that a Ni@SnNi composite possesses high lithium‐ion diffusion coefficient (≈10^−8^), which is much higher than those (≈10^−10^) reported previously, which can be mainly attributed to the unique flower‐like Ni@SnNi structure. In addition, the full cell performance (Ni@SnNi‐9h/graphite vs LiCoO_2_) with a capacity ratio of 1.13 (anode/cathode) is also tested. It is found that even at 2 C rate charging/discharging, the capacity retention at 100 cycles is still close to 89%. It means that Ni@SnNi‐9h is a promising anode additive for lithium‐ion batteries with high energy density and power density.

## Graphical abstract

In this work, a novel flower‐like Ni@SnNi structure is synthesized via a two‐step process design. The obtained Ni@SnNi composite exhibits an initial capacity of ≈693 mA h g^−1^ and a reversible capacity of ≈570 mA h g^−1^ after 300 charge/discharge cycles at 0.5 C, and maintains 450 mA h g^−1^ even at a high rate of 3 C.

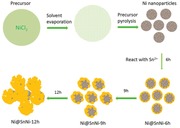

## 1 Introduction

From the essence of lithium‐ion batteries (LIBs), large current charging will not only lead to irreversible capacity fade quickly, but also cause a increase in internal resistance, leading to cell performance degradation.^1^ At present, the mainstream anode material of lithium‐ion battery in the market is graphite, and its lithium ion diffusion coefficient is less than 10^−10^ cm^2^ s^−1^ 2 and is difficult to adapt to the superquick charging in the furture.^3^ Hard carbon and soft carbon materials have also developed, but these carbon materials are generally with low coulombic efficiency and at high cost.^4^ Lithium titanate^5^ is recognized as a rapidly rechargeable anode material, but its gram capacity is low (175 mA h g^−1^) and lithiation potential is high (>1.55 v), resulting in low energy density. Similarly, a lot of fast‐charging anode materials, such as silicon oxide,^6^ titanium dioxide,^7^ nickel dioxide,^8^ niobium titanium, and various transition metal oxides^9^ are developing. Although these oxide anode materials have high lithium‐ion diffusion coefficient, they cannot avoid the defect of the low energy density of full cell, caused by the high lithiation potential. In addition, silicon and silicon‐based materials, the new generation of anode materials, have a relatively high capacity but their inherent poor conductivity and volume expansion in the process of charging and discharging lead to a gradual deterioration of cycle performance.^10^

Tin (Sn) has a high theoretical gravimetric capacity of 992 mA h g^−1^, thus is promising to be further anode materials.^11^, ^12^ However, the volumetric expansion of Sn during lithiation and delithiation is greater than 250%, which leads to pulverization of the active mass, resulting in poor cyclic stability.^13^ Nevertheless, LIB anodes composed of Sn‐based nickel (Ni) intermetallic composites, where Ni significantly enhances the capacity retention by forming an inactive framework to stabilize the electrode structure, has attracted considerable attention.^14^ Meanwhile, many reports have indicated that the size, morphology, and structure of SnNi intermetallic composites are important factors affecting performance, including capacity, charge/discharge rate, and cycle life.^15^, ^16^ Hence, it is of great significance to develop SnNi intermetallic composites with preferred structures, high specific capacity, fast‐charging performance, and, in particular, excellent cycling stability. It is also preferred that the synthesis process be simple and suitable for industrial production with low cost.

Herein, we designed and synthesized a novel Ni@SnNi core–shell structure via a two‐step process, consisting of the fabrication of Ni cores using spray pyrolysis followed by the formation of SnNi shells via a simple oxidation–reduction reaction between Ni and Sn^2+^. The obtained anode possesses high lithium‐ion diffusion coefficient (≈10^−8^), which is much higher than those (≈10^−10^) reported previously. As expected, the anodes formed of the obtained Ni@SnNi composite exhibited an initial capacity of ≈693 mA h g^−1^ and a reversible capacity of ≈570 mA h g^−1^ after 300 charge/discharge cycles at 0.5 C. Furtherly, it was proved that the flower‐like Ni@SnNi anodes can provide 450 mA h g^−1^ even at a high rate of 3 C. The cycle stability of the Ni@SnNi anode could be attributed to the unique flower‐like Ni@SnNi structure. And it is supposed that the excellent fast‐charging performance benefit from the lattice distortion of Sn crystal, which changes the size of ions diffusion channels and the coulomb force between lithium‐ion and surrounding lattice as well as, and fundamentally improve the ion diffusion coefficient.

## 2 Results and Discussion

**Figure 1:**
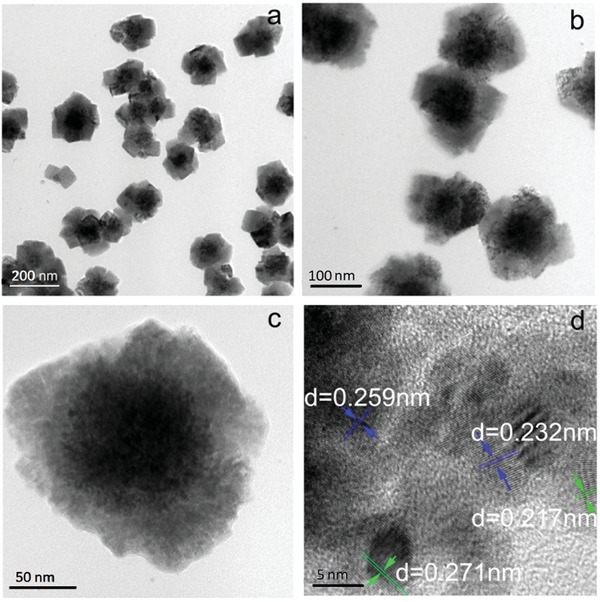
TEM images of Ni@SnNi‐9h core–shell composite particles.

**Figure 2:**
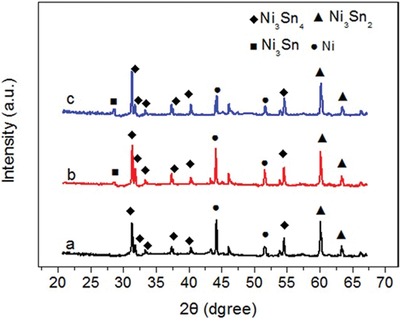
XRD curves of a) Ni@SnNi‐6h, b) Ni@SnNi‐9h, and c) Ni@SnNi‐12h anodes.

Spray pyrolysis is a typical method to prepare metal or metal oxides particles with porous and microspheres structure.^11^, ^17^ The sketch of the aerosol spray pyrolysis apparatus was shown in Figure S1 of the Supporting Information, according to other work.^18^ NiCl_2_ was decomposed to Ni atoms under the N_2_/H_2_ environment.^11^ The obtained Ni atoms form Ni nuclei first and grow through diffusion–coalescence processes. During this stage, the Ni atoms in molten state form Ni microspheres. Transmission electron microscopy (TEM) images of Ni@SnNi‐9h particles are shown in **Figure** **1**, which demonstrate Ni cores with diameters of ≈100 nm and NiSn shells with thicknesses of 50 nm. The elements mapping further manifest the flower‐like Ni@NiSn structure as shown in Figure S2 of the Supporting Information. It is really worth noting that the spacing of adjacent lattice fringes for the Ni@SnNi particles are 0.217, 0.232, 0.259, and 0.271 nm, respectively, as indicated by the selected area diffraction image in Figure 1d. These lattice fringes are different from these of pure metallic Sn and Ni where Sn (301), Sn (301), Sn (211), Ni (111), and Ni (200) correspond to 0.29, 0.16, 0.20, 0.20, and 0.18 nm,^19^ respectively. This can be attributed to the formation of a Sn/Ni intermetallic compound through a galvanic displacement process between Ni and Sn^2+^. In this Sn/Ni intermetallic compound, the interference of Ni atoms resulted the lattice distortion of Sn crystal.^20^ To detect the lattice distortion, in addition to TEM, X‐ray diffraction (XRD) is also a persuasive characterization measure.^21^ As shown in **Figure** **2**, the diffraction patterns of the flower‐like Ni@NiSn are demonstrated at *2θ* = 28.5°, 31.2°, 31.8°, 33.3°, 37.4°, 40.2°, 44.3°, 46.3°, 51.7°, 54.6°, 60.3°, and 63.4°. Among the diffraction peaks, pure Ni phase structure (JCPDS Card No. 04‐0850) is evidenced by the peaks appearing at 44.3° and 51.7°. While other diffraction peaks correspond to the intermetallic compound phase of Ni_3_Sn_4_, Ni_3_Sn_2_, and NiSn overlaping each other.^22^ The formation of intermetallic compound originated from the interference Ni atoms into Sn lattice.^23^ Since lattice distortion is an intrinsic characteristic of the formation of intermetallic compound,^24^ it is reconfirmed that the interference of Ni atoms into Sn generates lattice distortion of pure Sn crystal, which are consistent with the results as shown in previous TEM images.

**Figure 3:**
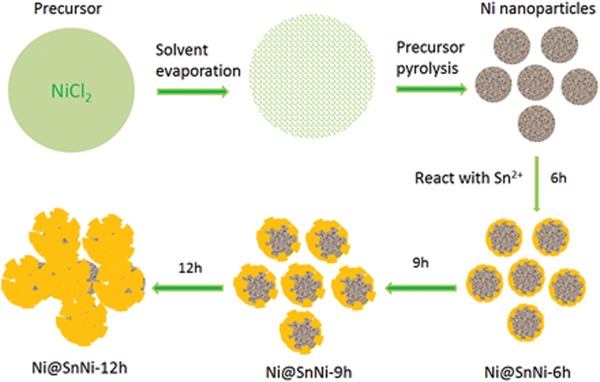
Formation mechanism of Ni@SnNi core–shell composite particles.

**Figure 4:**
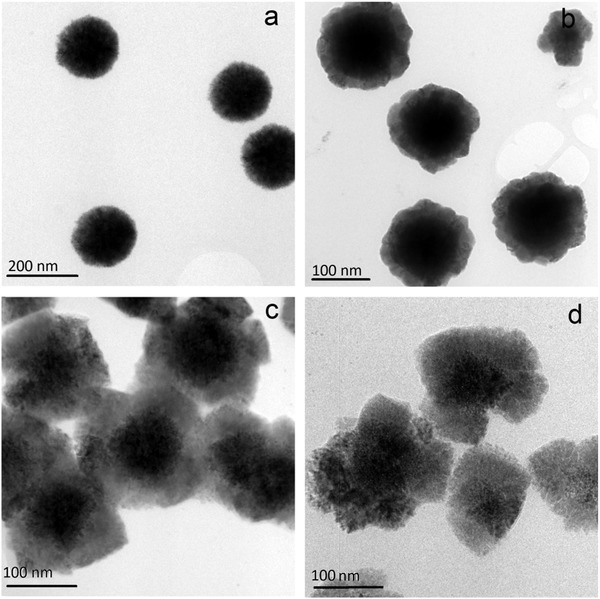
TEM images of a) Ni cores, b) Ni@SnNi‐6h, c) Ni@SnNi‐9h, and d) Ni@SnNi‐12h composites.

The formation mechanism of Ni@NiSn structure is illustrated in **Figure** **3**, where the nanosized Ni cores are first synthesized, and then galvanic displacement between Ni and Sn^2+^ occurs slowly, generating the SnNi shell. The reaction progress is also shown in **Figure** **4**. We note that, when the galvanic displacement reaction time was 6 h, the thickness of the SnNi shell was about 20 nm, while a reaction time of 9 h provided a shell thickness of about 50 nm. Simultaneously with this process of shell growth, the diameter of the Ni core was accordingly decreased gradually. This growth process is particularly evident for a reaction time of 12 h, where nearly the entire Ni core material has been converted to SnNi shell material. The corresponding Sn contents of the Ni@SnNi‐6h, Ni@SnNi‐9h, and Ni@SnNi‐12h particles were estimated as 54, 70, and 86 wt%, respectively, according to the mass of obtained production, based on oxidation–reduction reaction equation of Ni and Sn^2+^.

**Figure 5:**
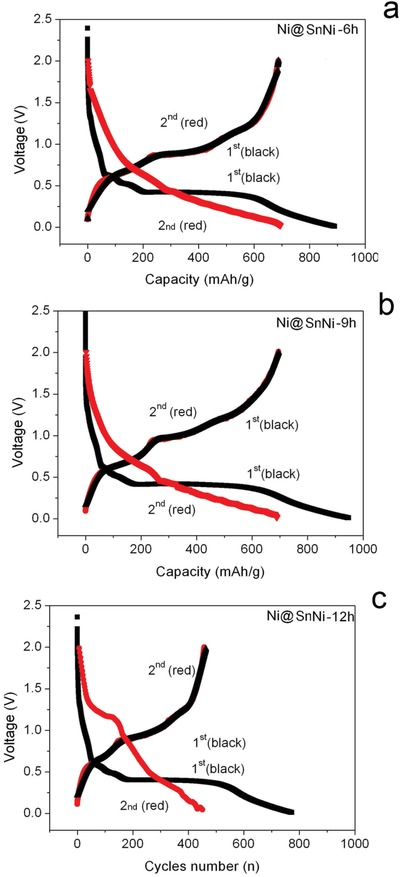
Charge/discharge curves at 0.5 C for a) Ni@SnNi‐6h, b) Ni@SnNi‐9h, and c) Ni@SnNi‐12h anodes.

**Figure 6:**
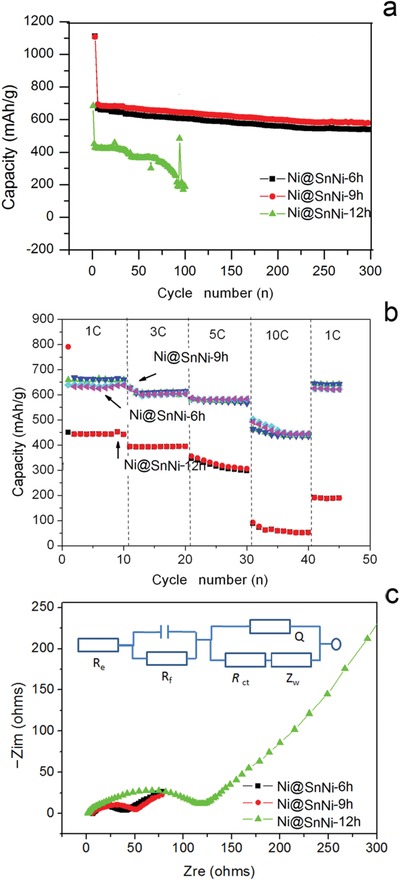
Cycling performance at a) 0.5 C rate, b) rate performance, and c) Nyquist plots of Ni@SnNi‐6h, Ni@SnNi‐9h, and Ni@SnNi‐12h anodes, respectively. The equivalent circuit model used to simulate the spectra is presented as an inset in (c).

**Figure** **5** shows the voltage profiles of the 1st and 2nd cycles at a current density of 0.5 C in a voltage window of 0.02–2.0 v at room temperature. The results indicate that the respective first discharge/charge capacities of the Ni@SnNi‐6h and Ni@SnNi‐9h anodes were 900/693 and 900/703 mA h g^−1^, with first columbic efficiencies of 78.1% and 80%, respectively. In sharp contrast to the Ni@SnNi‐6h and Ni@SnNi‐9h anodes, the initial discharge/charge capacities of the Ni@SnNi‐12h anode were 780/453 mA h g^−1^, with a first columbic efficiency of 58.0%. The initial irreversible loss of all samples is related to the formation of a solid electrolyte interphase (SEI) layer on the anode surface,^15^, ^25^ The Ni in the Ni@SnNi‐6h and Ni@SnNi‐9h can effectively buffer the volumetric change in the anode during the first charge/discharge process, which benefits from the formation of a stable SEI film in the first cycle. In subsequent cycles, as shown as **Figure** **6**a, the reversible capacity of the Ni@SnNi‐6h and Ni@SnNi‐9h anodes was maintained above 570 mA h g^−1^, even at the 300th cycle. However, the Ni in the Ni@SnNi‐12h anode, which is not in a core–shell structure, cannot effectively accommodate the large volumetric changes, which leads to constant cracking of the SEI layer, with a portion of the Ni@SnNi‐12h anode transforming into inactive Li*_x_*Sn/Ni compounds during the charge/discharge process (as shown in Figure S3, Supporting Information). As a result, the capacity of the Ni@SnNi‐12h anode is only about 100 mA h g^−1^ at the 100th cycle. In order to certify the stability related volumetric changes, the SEM images of Ni@SnNi‐12h and Ni@SnNi‐9h after long cycling are contrastively checked. From Figure S4 of the Supporting Information, we can see after long cycling of 100 cycles, the particles of Ni@SnNi‐9h are almost integrated while the particles of Ni@SnNi‐12h have some visible cracks and pulverization resulting from volumetric changes during intercalation/deintercalation of lithium ions. Furthermore, the rate capabilities of anodes formed with the different Ni@SnNi composites were tested with charge/discharge rates increased from 1 to 10 C, as shown in Figure 6b. As the rate was increased to 1 C the Ni@SnNi‐6h anode demonstrated a small decrease in discharge capacity from 660 to 605 mA h g^−1^. When the rate was increased to 3 C, the discharge capacity decreased to 447 mA h g^−1^, which represents a high capacity retention of 67.7%. The Ni@SnNi‐9h anode demonstrated even better cycling performance, where the capacity retention was around 70.3% when the rate was increased to 3 C. However, the Ni@SnNi‐12h anode demonstrated a capacity retention of only 15.8% when the rate was increased to 3 C.

Lithium‐ion diffusion coefficient is one of the most important parameters used to evaluate the performance of electrode materials, especially fast‐charging performance. It is usually recognized that the higher is the lithium‐ion diffusion coefficient and the better is the fast‐charging performance. The diffusion coefficient of lithium ions (*D*_Li+_) in the Ni@NiSn electrode can be calculated via a typical equation^26^

(1) $$D_{{\text{Li}}^{\text{+}}}=\frac{R^{2}T^{2}}{2A^{2}n^{4}F^{4}C^{2}\sigma^{2}}$$

where *R* is the gas constant, *T* is the tested absolute temperature under test conditions, *A* is the surface area of the working electrode, *n* is the number of electrons per molecule involved in the electronic transfer reaction, *F* is Faraday constant, *C* is the Li^+^ concentration in the working electrode, and the σ is the slope of the plots of *Zw* against ω^−1/2^.^27^ Electrochemical impedance spectroscopy (EIS) measurements with the corresponding equivalent circuit are shown in Figure 6c. Generally, the plots consist of a high‐frequency semicircle and a medium‐frequency semicircle, which overlap each other, and a gradient line in the low frequency region.^28^ The values of as‐prepared flower‐like Ni@NiSn samples for *R*_e_, *R*_f_, *R*_ct_, and *R* total are exhibited in Table S1 of the Supporting Information, which is simulated from EIS data via the equivalent circuit as shown in Figure 6c inset. Comparisons among these three anode samples indicates that the values of *R*_e_ and *R*_ct_ are not significantly different. However, the values of *R*_f_ for Ni@SnNi‐6h (22.3 Ω) and Ni@SnNi‐9h (17.2 Ω) anodes are obviously lower than that of *R*_f_ (320.0 Ω). Accordingly, the Ni@SnNi‐6h and Ni@SnNi‐9h anodes demonstrated nearly higher *D*_Li_^+^ values, which is 3.8 × 10^−8^ and 8.7 × 10^−8^ cm^2^ s^−1^, respectively, which are much higher than those reported previously.^29^ Overall, to achieve a high Li^+^ diffusion coefficient, the size of Li^+^ diffusion channels in the crystal should match the size of the Li^+^ radius. Moreover, the bonding force between Li^+^ and crystal framework should be weak, so as to reduce the ion transport resistance.^30^ In our case, it is supposed that the interference of Ni atoms causes the lattice distortion of Sn crystal,^20^ which changes the size of ions diffusion channels and the coulomb force between ions and surrounding lattice as well as, and fundamentally improve the Li^+^ diffusion coefficient. In addition, nanolevel structure also contribute high Li^+^ diffusion coefficient, which provides a short charge transfer path. The resulted Ni@SnNi‐9h composite has more suitable structure for Li^+^ transmission and the highest Li^+^ diffusion coefficient. Thus far, no other previously reported Sn‐based anode material has demonstrated the above three properties simultaneously.^16^, ^17^, ^18^, ^19^

**Figure 7:**
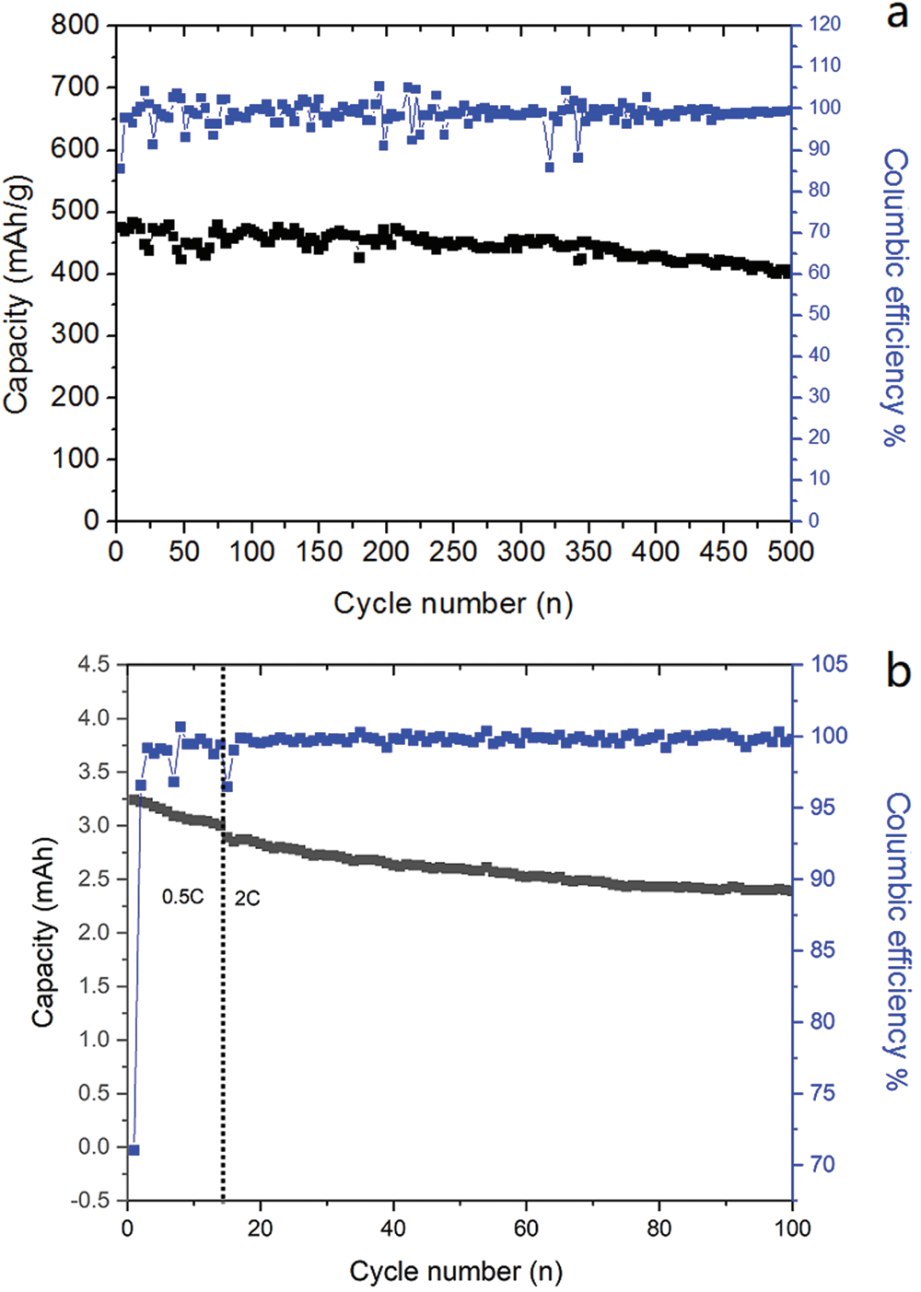
The cycling performance at a) 0.5 C rate of Ni@SnNi‐9h/graphite, and charge/discharge curves at b) 0.5 and 2 C rate of full cell (Ni@SnNi‐9h/graphite vs LCO).

Finally, for the purpose of application, the performance of hybrid of Ni@SnNi‐9h and commercial graphite (Ni@SnNi‐9h/graphite) was tested in half cells. It is surprised that the capacity of this hybrid is about 460 mA h g^−1^ with a higher first columbic efficiency of 85%. The capacity retention keeps to above 87% after 500 cycles as shown in **Figure** **7**a. Further, the full cell performance with a capacity ratio of 1.13 (anode/cathode) was also tested. As shown in Figure 7b, we can see even at 2 C rate charging/discharging, the capacity retention at 100 cycles is still close to 89%. It means that Ni@SnNi‐9h is promising anode additive for lithium‐ion batteries with high energy density and power density.

## 3 Conclusions

In conclusion, we developed a two‐step route, including spray pyrolysis followed by a simple oxidation–reduction reaction, to synthesize Ni@SnNi core–shell intermetallic composites with high Li^+^ diffusion coefficient. Anodes formed of the obtained Ni@SnNi composites demonstrated high capacity, long cycle‐life, and good fast‐charging performance, which represents a promising anode material and anode additive. It is proved that Ni@SnNi‐9h is a promising anode additive for lithium‐ion batteries with high‐energy density and power density. Moreover, the synthesis process is easily scaled; thus, the synthesis method is appropriate for industrial‐scale production.

## 4 Experimental Section

*Preparation of Ni@NiSn Composite*: In the typical spray pyrolysis process as shown in Figure S1 of the Supporting Information, 23.8 g of NiCl_2_·6H_2_O was first dissolved into 400 mL of ethanol. Second, the obtained precursor solution consisting of Ni ions was aerosolized. Third, the aerosol underwent pyrolysis via a tubular furnace at 1000 °C with a residence time of ≈1 s to produce Ni nanoparticles. Finally, the formed solid particles were collected on a DTTP Millipore filter with a pore size of 0.4 µm. To prepare the Ni@SnNi core–shell structure, three obtained Ni nanoparticles with the same mass of 1.47 g was dissolved with double distilled water (100 mL), respectively, followed by the addition of 100 mL of tin (II) chloride dehydrate (SnCl_2_) with a concentration of 0.18 mol L^−1^. The obtained three mixtures were stirred for 6, 9, and 12 h, respectively, at room temperature with sonication treatment. The solution was centrifuged and washed with double distilled water three times to obtain solid Ni@SnNi particles. The resulting particle samples are respectively denoted herein as Ni@SnNi‐6h, Ni@SnNi‐9h, and Ni@SnNi‐12h according to the reaction time. For practical purposes, the Ni@SnNi‐9h and commercial graphite with mass ratio of 0.22:0.78 (Ni@SnNi‐9h:graphite) were mixed, and named as Ni@SnNi‐9h/graphite.

*Materials Characterization*: TEM images were obtained using a high‐resolution JEOL 2012F instrument. The structure of the intermetallic compound was measured by XRD (X'pert PRO, Panalytical, the Netherlands) with a Cu Kα radiation and a scanning rate of 10° min^−1^ in a range of 2θ values from 10° to 70°.

*Electrochemical Characterization*: Half cells tests were performed on 2032 coin‐type cells using the resulting samples as cathodes and lithium foil as anodes microporous polypropylene as the separator and 1 m LiClO_4_ in ethylene carbonate/dimethyl carbonate (1:1, volume ratio) as electrolyte with 2% (v:v) fluoroethylene carbonate as additive. EIS was conducted in the 100 kHz to 0.01 Hz frequency range with a perturbation amplitude of 5 mV. Full cells tests were performed on 2032 coin‐type cells using the resulting hybrid of Ni@SnNi‐6h/graphite as anode with an active material mass loading of 6.2 mg cm^−2^ and commercial LiCoO_2_ cathode with a ratio anode capacity/cathode capacity = 1.13. Electrochemical performance was tested on a Land CT2001A system in the range of 2.7 and 4.2 v (vs Li/Li^+^) with 0.5 C rate and 3 C rate, respectively.

## Conflict of Interest

The authors declare no conflict of interest.

## Supporting information

Supporting Information
