# Supplementary material for: Synthesis of Ni@NiSn Composite with High Lithium‐Ion Diffusion Coefficient for Fast‐Charging Lithium‐Ion Batteries
Source: Glob Chall. 2019 Nov 22;4(3):1900073. doi: 10.1002/gch2.201900073 (PMC7050083; doi:10.1002/gch2.201900073)
Supplement: Supplementary file 1 — Supporting Information [file GCH2-4-1900073-s001.pdf]

Copyright WILEY-VCH Verlag GmbH & Co. KGaA, 69469 Weinheim, Germany, 2019.

Supporting Information

for *Global Challenges,* DOI: 10.1002/gch2.201900073

Synthesis of Ni@NiSn Composite with High Lithium-Ion Diffusion Coefficient for Fast-Charging Lithium-Ion Batteries

*Hong Zhao, Junxin Chen, Weiwei Wei, Shanming Ke, Xierong Zeng, Dongchu Chen, and Peng Lin**

Synthesis of Ni@NiSn composite with high lithium-ion diffusion coefficient for fast-charging lithium-ion batteries

Hong Zhao[1,2,3#], Junxin Chen[1#], Weiwei Wei[1], Shanming Ke[1], Xierong Zeng[1], Dongchu Chen[2], Peng Lin[1]*

1. *Shenzhen Key Laboratory of Special Functional Materials & Shenzhen Engineering Laboratory for Advanced Technology of Ceramics, College of Materials Science and Engineering, Shenzhen University, Shenzhen 518060, P. R. China. E-mail: lin.peng@szu.edu.cn*
2. *School of materials science and energy engineering, Foshan university*
3. *Department of Mechanical and Aerospace Engineering, Hong Kong University of Science and Technology*

# These authors contributed equally to this work.

Additional Information:

Competing Financial Interests statement: *The authors have declared that no competing interests exist.*

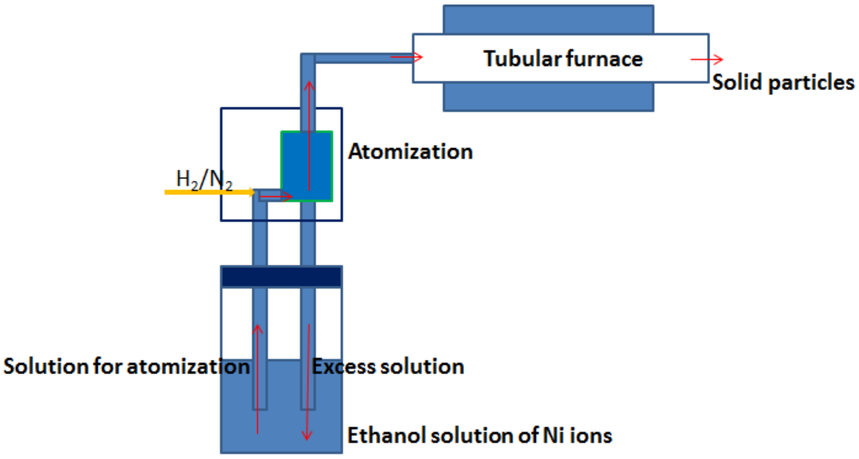

Figure S1. Sketch of the aerosol spray pyrolysis apparatus [1].

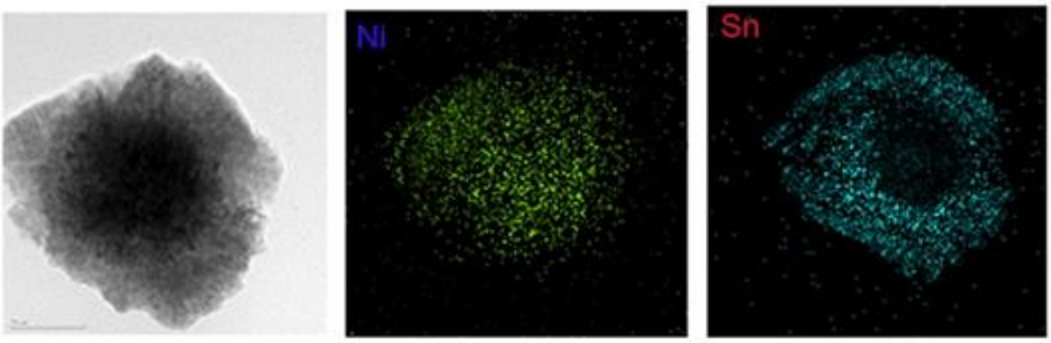

Figure S2. The elements mapping of the flower-like Ni@NiSn-9h.

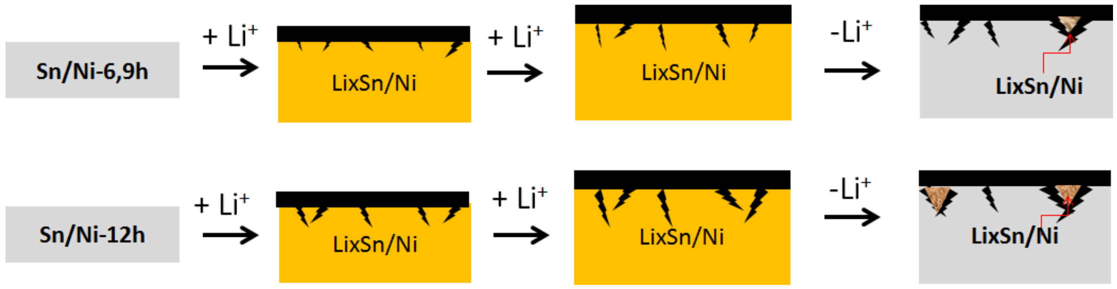

Figure S3. Model for the SEI film formation on flower-like Ni@NiSn compound according to reference [2].

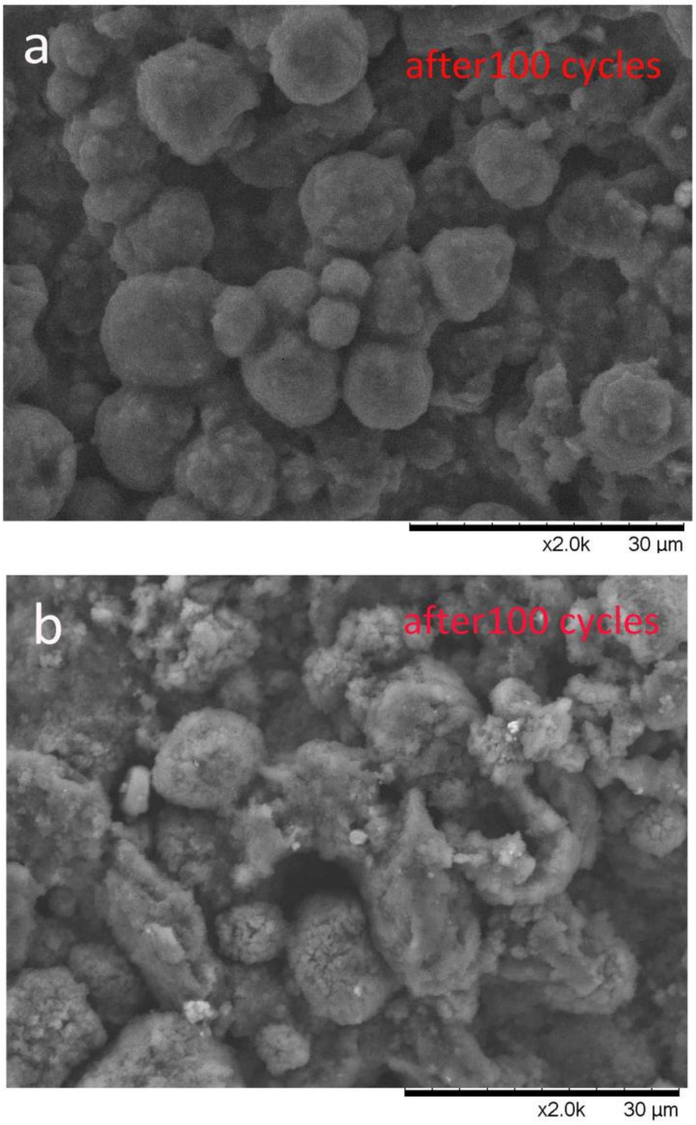

Figure S4. SEM images of Ni@SnNi-9h (a) and Ni@SnNi-12h (b) after cycling of 100cycles.

Table 1 The simulated parameters of EIS using the equivalent circuit for flower-like Ni@NiSn compound.

Samples	R_e [ohm]	R_{ct} [ohm]	R_f [ohm]	R_{total} [ohm]
SnNi@Ni-6h	2.7	152.1	22.3	177.1
SnNi@Ni-9h	3.9	178.3	17.2	199.4
SnNi@Ni-12h	5.1	143.6	320.0	468.7

Table 2 Warburg factor and diffusion coefficient ions under different discharge states for flower-like Ni@NiSn compound.

Samples	1.0V		1.5V	
	$\sigma/\Omega s^{-1/2}$	D_{Li^+}/ cm^2s^{-1}	$\sigma/\Omega s^{-1/2}$	D_{Li^+}/ cm^2s^{-1}
Ni@NiSn-6h	102.1	$3.8*10^{-8}$	154.5	$1.2*10^{-8}$
Ni@NiSn-9h	114.0	$8.7*10^{-8}$	124.7	$1.8*10^{-8}$
Ni@NiSn-12h	365.2	$1.1*10^{-9}$	456.0	$0.2*10^{-9}$

References:

[1] C. M. Sorensen, Q. Li, H. K. Xu, Z. K. Tang, K. J. Klabunde and G. C. Hadjipanayis, **Aerosol Spray Pyrolysis Synthesis Techniques**, in Nanophase Materials, Springer, 1994, pp. 109-116.

[2] M. Wachtler, J. O. Besenhard, M. Winter, **Tin and tin-based intermetallics as new anode materials for lithium-ion cells.** Journal of Power Sources, 94 (2001) 189-193
